# Optimizing Cellulase—*Limosilactobacillus fermentum* ZC529 Synergy Fermentation for Preserving *Macadamia integrifolia* Pericarp’s Potential Use as Antioxidants

**DOI:** 10.3390/antiox13070783

**Published:** 2024-06-27

**Authors:** Chen Zhang, Haibo Huang, Bifan Liu, Xiongzhuo Tang, Bi’e Tan, Qian Jiang, Yulong Yin

**Affiliations:** 1 Animal Nutritional Genome and Germplasm Innovation Research Center, College of Animal Science and Technology, Hunan Agricultural University, Changsha 410128, China; zhch@hunau.edu.cn (C.Z.); bietan@hunau.edu.cn (B.T.); 2 Institute of Yunnan Circular Agricultural Industry, Pu’er 665000, China; 3 Yuelushan Laboratory, Changsha 410128, China; 4 Institute of Subtropical Agriculture, Chinese Academy of Sciences, Changsha 410125, China

**Keywords:** *Macadamia integrifolia* pericarp, enzyme–bacteria synergy fermentation, response surface methodology (RSM), antioxidant, *Drosophila melanogaster*

## Abstract

*Macadamia integrifolia* pericarps (MIP) are byproducts of nut production which are rich in natural antioxidant compounds, making them an excellent source for extracting bioactive compounds. However, the antioxidant compounds in MIP are easily oxidized under natural storage conditions, resulting in significant biomass loss and resource wastage. To preserve the potential of MIP to be used as an antioxidant product, we employed cellulase and *Limosilactobacillus fermentum* ZC529 (*L.f* ZC529) fermentation and utilized response surface methodology to optimize the fermentation parameters for mitigating the antioxidant loss. Total antioxidant capacity (T-AOC) was used as the response variable. The fermented MIP water extract (FMIPE) was obtained via ultrasound-assisted extraction, and its biological activity was evaluated to optimize the best fermentation conditions. Results indicated that a cellulase dosage of 0.9%, an *L.f* ZC529 inoculation size of 4 mL/100 g, and a fermentation time of 7 days were the optimal conditions for MIP fermentation. Compared to spontaneous fermentation, these optimal conditions significantly increased the total phenolic and total flavonoid contents (*p* < 0.05). T-AOC was 160.72% increased by this optimal fermentation (*p* < 0.05). Additionally, supplementation with varying concentrations of FMIPE (6.25%, 12.5%, and 25%) increased the T-AOC, SOD activity, and GSH content, and reduced MDA levels of the oxidative-stressed *Drosophila melanogaster* (*p* < 0.05). Moreover, 12.5% and 25% of FMIPE treatments elevated CAT activity in the *Drosophila melanogaster* (*p* < 0.05). The effects of FMIPE on GSH and MDA in *Drosophila melanogaster* were equivalent to the 0.5% vitamin C (Vc) treatment. In summary, synergistic fermentation using cellulase and *L.f* ZC529 effectively preserves the antioxidant activity of the MIP, offering a simple, eco-friendly method to promote the utilization of MIP resources.

## 1. Introduction

*Macadamia integrifolia* pericarp (MIP) is the green outer skin of the macadamia (*Macadamia integrifolia* Maiden and Betche). It is an agricultural byproduct generated during the processing of the nut, accounting for approximately 50% of the fresh fruit weight [1]. These byproducts are typically considered low-value waste [2]. Research indicates that nut pericarps are a rich source of phenolic compounds [3], making MIP an important raw material for recovering phenolic compounds. Research has found that the MIP is rich in various natural antioxidant compounds (Table 1), including tannins 1.61 ± 0.55% [4], total phenols 18.36 ± 0.32 mg/g [5], total flavonoids 7.33 ± 2.63 mg/g [6], and other antioxidants such as 4-hydroxybenzyl alcohol [7]. In addition, studies by Shi Rui et al. [8] and Dailey A et al. [9] have discovered arbutin, helicid (4-formylphenyl β-D-glucopyranoside), and procyanidin in MIP.

Research indicates that the ethanol extract of MIP has an IC50 of 4.13 mg/L against DPPH and 112.94 mg/L against ABTS^+^, and its total antioxidant capacity is approximately 1.7 times that of a water-soluble derivative of vitamin E (Trolox) [6]. The ethyl acetate extract of MIP has the strongest scavenging ability against DPPH and ABTS^+^, with IC50s of 0.67 and 0.05 mg/mL, respectively. It has also been confirmed to possess a stronger reducing ability (IC50 = 0.09 mg/mL) compared to the rutin standard (IC50 = 0.68 mg/mL), as measured by FRAP assay [10]. And, the *n*-butanol extract of MIP exhibits a strong scavenging ability against superoxide anion (O_2_^●−^), with an IC50 = 0.08 mg/mL. At the same concentration, it surpasses the rutin standard (IC50 = 0.18 mg/mL) in efficacy [10]. Somwongin et al. [11] pointed out that the water extract of MIP obtained through ultrasound-assisted extraction possesses the strongest antioxidant and anti-skin-aging activities. It has the highest total phenolic content, exhibiting antioxidant activity comparable to vitamin C (Vc) and trolox. Additionally, its anti-skin-aging activity is comparable to that of epigallocatechin-3-gallate and oleanolic acid. However, as living plant tissues, the MIP undergoes processes such as roasting, pressing, and peeling after harvesting. These processes subject the tissues to physical stress, leading to the substantial accumulation of reactive oxygen species (ROS) and a substantial loss of antioxidant components in a short period of time [12], resulting in a significant decrease in its usable value and being treated as waste and disposed of through landfilling [13]. MIP is an excellent source for extracting bioactive compounds, but it is far from being fully utilized [14].

Lactic acid bacteria (LAB), as natural fermenting agents, are widely used in the fermentation of food and feed. Fermentation by LAB is an effective strategy to enhance the levels and biological characteristics of bioactive components in agricultural byproducts [15]. Studies have shown that LAB fermentation can protect the bioactive substances in plant tissues, promote the release of phenolic compounds, and enhance the antioxidant activity of fermented products [16]. LAB can also increase the content of antioxidant compounds in fermentation products through biotransformation. For example, Li et al. increased the content of phenolic acids and isoflavones by fermenting soybeans with *Lactobacillus casei*, and the antioxidant activity of whole-soybean flour was significantly improved [17]. Kuo et al. reported that fermenting *Chenopodium formosanum* Koidz. with *Lactobacillus plantarum* BCRC 11697 could enhance the DPPH and ABTS^+^ free-radical-scavenging abilities [18]. *Limosilactobacillus fermentum* (*L.f*) is a LAB with antioxidant, antibacterial, and immune-modulating activities [19,20], which can be used to ferment grains and other plant materials and dairy products and make the products rich in bioactive compounds with antioxidant and anti-inflammatory properties [20]. It is considered a bacterial species with potential for food preservation and biomedical applications [21].

However, there is currently no information available on using *L.f* to enhance the antioxidant activity of MIP. When fermenting MIP with *L.f*, the *L.f* inoculation size, the cellulase dosage, and the fermentation time will determine the final antioxidant performance of the fermented product [1,22]. Therefore, this study aims to explore and determine the optimal antioxidant fermentation process of MIP using *L.f*. We will analyze the presence of bioactive compounds (total phenolic content, flavonoid content) in the fermented product, clarify the value-added effect of *L.f* fermentation on MIP, and further assess the benefits of the fermented product under optimal fermentation conditions using an oxidative stress *Drosophila* model.

## 2. Materials and Methods

### 2.1. Materials

After MIP (Lancang County, Yunnan Province, China, 22°33′ N, 99°55′ E) was collected at the harvest stage of *Macadamia integrifolia*, it was crushed into pieces smaller than 1 cm^2^ and thoroughly mixed before being randomly sampled for fermentation. *Limosilactobacillus fermentum* ZC529 (*L.f* ZC529) was isolated, identified, and preserved from fresh MIP and Diannan small-ear (DSE) pigs’ intestines. Cellulase, enzyme activity ≥ 2 × 10^4^ U, was purchased from Beijing Challenge Biotechnology Co., Ltd., Beijing, China; gstD-GFP type *Drosophila melanogaster* was provided by the Animal Nutrition Genome and Germplasm Innovation Center of Hunan Agricultural University, Changsha, China.

### 2.2. Optimization Design of the Fermentation Process

#### 2.2.1. Experimental Design for Single-Factor

Study on the impact of cellulase dosage (A, 0.15%, 0.3%, 0.6%, 0.9%, 1.2%), *L.f* ZC529 (10^8^ CFU/mL) inoculation size (B, 1, 2, 3, 4, 5 mL/100 g), and fermentation time (C, 1, 3, 5, 7, 9 days) on the antioxidant capacity of fermented MIP, respectively, was conducted. MIP fermentation was carried out using fermented bags (290 × 230 mm^2^, with a one-way valve; Wenzhou Wangting Packaging Co., Ltd., Zhejiang, China) with 500 g of material per bag. The bags were vacuum-sealed and stored at 30 ± 2 °C. The total antioxidant capacity (T-AOC) was assessed by the T-AOC Assay Kit (Nanjing Jiancheng Bioengineering Institute, Nanjing, China).

#### 2.2.2. Experimental Design for Optimization

Response surface methodology (RSM) was used to optimize the fermentation of MIP using the software Design Expert (v 13.0, Stat-Ease Inc., Minneapolis, MN, USA). A three-level, three-factor (Table 2) Box–Behnken design was used with three central points replicated in the design of the experimental conditions, based on preliminary single-factor tests [23].

Seventeen experimental runs were performed following a central composite design. The different combinations of the process parameters were studied, and the main responses achieved in each fermentation run are summarized in Table 3. The correlation between the independent and dependent variables was explained through the second-order polynomial model [15] outlined in the following Equation (1).

(1) $$Y=\beta_{0}+\sum_{i=1}^{k}\beta_{i}X_{i}+\sum_{i=1}^{k}\beta_{ij}X_{i}^{2}+\sum_{\begin{array}{c} i=1\\ i<j \end{array}}^{k-1}\sum_{j=i+1}^{k}\beta_{ij}X_{i}X_{j}$$

where Y stands for a predicted response (total antioxidant capacity, T-AOC); X_i_ and X_j_ are independent variables affecting the responses Y; β_0_, β_i_, β_ii_, and β_ij_ are the regression coefficients for intercept, linear, quadratic, and interaction terms, respectively; and k is the number of variables. One model was generated for each dependent variable.

The three independent variables were assigned as A (cellulase dosage, %), B (inoculation size, mL/100 g), and C (fermentation time, day). Thus, the function containing these three independent variables [9] is expressed as follows (2):

(2) $$Y=\beta_{0}+\beta_{1}A+\beta_{2}B+\beta_{3}C+\beta_{12}AB+\beta_{13}AC+\beta_{23}BC+\beta_{11}A^{2}+\beta_{22}B^{2}+\beta_{33}C^{2}$$

where Y is the response; A, B, and C are the independent factors; β_0_ is the intercept coefficient; β_1_, β_2_, and β_3_ are the linear interaction coefficients; β_13_, β_12_, and β_23_ are the squared effects terms; and β_11_, β_22_, and β_33_ are the interaction coefficients.

### 2.3. Determination of Total Antioxidant Capacity, Total Phenolic Content, and Flavonoid Content

Based on the results of Section 2.2, MIP was processed under optimal fermentation conditions, designated as the *L.f* group. Simultaneously, MIP was treated with sterile water of the same volume as the *L.f* ZC529 inoculation size, designated as the H_2_O group. Both groups were fermented at 30 °C for the same duration. Unfermented MIP (stored at −20 °C) was designated as the control group. Following the method described by Somwongin et al. [11], the samples were subjected to ultrasound-assisted water extraction (37 kHz, 80 W, 10 min) using deionized water at a weight ratio of 1:5. The total phenolic and total flavonoid contents were determined using kits from ZCIBIO Technology Co., Ltd. Shanghai, China, while the antioxidant capacity was assessed using a T-AOC assay kit from Nanjing Jiancheng Bioengineering Institute.

### 2.4. Feeding and Treatment of Flies

The *Drosophila* transgenic reporter strain (*gstD1-GFP*) was maintained on an instant potato medium and 3–5-day-old males were collected for further experiment. Subsequently, oxidative stress was induced by feeding flies with a 200 μL mixture of sucrose solution (5%) containing 0.3% H_2_O_2_. The oxidative stress model flies were randomly divided into 5 groups and maintained in plastic vials placed with a sterile dry paper disk (φ = 18 mm, δ = 0.3 mm) at the bottom. Each vial contains 20 flies, with 5 vials per group (n = 100). Following the method described by Somwongin et al. [11], ultrasound-assisted water extraction was performed on the fermented MIP produced under optimal fermentation conditions to prepare 6.25%, 12.5%, and 25% fermented MIP water extracts (FMIPE), respectively. On this basis, add 5% sucrose to each extraction solution and record them as low, medium, and high groups (Table 3). Mix thoroughly and serve as food for the flies. Normal feed (a 5% sucrose solution) served as the negative control (H_2_O_2_ group), while feed supplemented with Vc (0.5% Vc solution containing 5% sucrose) served as the positive control (Vc group). Healthy flies fed with normal feed served as the blank control (control group). All flies were raised under conditions of 55 ± 5% relative humidity, a synchronized 12 h light-dark cycle, and a temperature of 25 °C.

The flies were fed with food through melting-point capillary tubes (0.5 × 100 mm), and the paper disk was replaced every 12 h. The Flies were fed using capillary tubes for 5 days, and the survival rates of each group were recorded. Twenty flies were randomly selected from each treatment group, weighed, and transferred to sterile homogenizers after rapid freezing in liquid nitrogen. Each 0.1 g sample was mixed with 1 mL of pre-chilled PBS to prepare whole-organism homogenates. After centrifugation at 8000× *g* for 10 min at 4 °C, the supernatant was collected. T-AOC, the activities of superoxide dismutase (SOD), and catalase (CAT) activity, and the levels of reduced glutathione (GSH) and malondialdehyde (MDA) in the flies were determined according to the instructions provided with the assay kits (Nanjing Jiancheng Bioengineering Institute, Nanjing, China).

### 2.5. Statistical Analysis

An analysis of variance (ANOVA) was used to compare the experimental and anticipated values’ responses using SPSS 16.0. Evaluate the response using a second-order polynomial equation, and then, use multiple regression analysis to fit the data into the equation. With *p* < 0.05 signifying significant differences, the data are reported as mean ± standard deviation (SD). Plot and output the results with Graph Pad Prism 8.0.2.

## 3. Results

### 3.1. The Results of the Single-Factor Experiments

**Figure 1:**
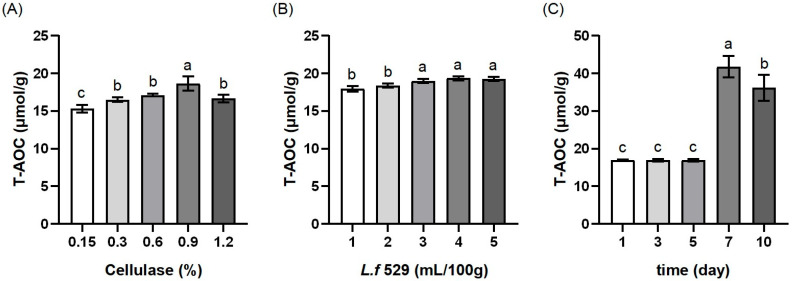
Effects of different independent variables on the T-AOC of MIP. *Note*: (**A**), (**B**), and (**C**), respectively, represent the effects of cellulase dosage, *L.f* ZC529 inoculation size, and fermentation time on the T-AOC (total antioxidant capacity) of MIP. Different lowercase letters indicate significant differences among treatment groups (*p* < 0.05).

The influence of cellulase dosage, *L.f* ZC529 inoculation size, and fermentation time on the T-AOC of MIP are shown in Figure 1. The T-AOC showed an increasing trend with increasing the cellulase dosage, followed by a decrease. When the cellulase dosage was 0.9% (*p* < 0.05), the T-AOC was highest, reaching 18.65 μmol/g (Figure 1A). With the increase in the *L.f* ZC529 inoculation size, the T-AOC increased. When the inoculation size was 4 mL/100 g, the total antioxidant capacity of MIP reached its maximum value, and then slightly decreased with further increases in the *L.f* ZC529 inoculation size (Figure 1B). As the fermentation time increased, the total antioxidant capacity of MIP increased, reaching its maximum at 7 days (*p* < 0.05) (Figure 1C). With a further increase in fermentation time, the T-AOC no longer increases. Therefore, in subsequent experiments, the cellulase dosage was optimized between 0.6% and 1.2%, the *L.f* ZC529 inoculation size was optimized between 3 mL/100 g and 5 mL/100 g, and fermentation time was optimized between 5 days and 9 days.

### 3.2. Modeling of the MIP Fermentation Process

According to the experimental results of the single-factor experiments, the response surface experiment was designed with the T-AOC as the dependent variable. The coding values of the three factors and the corresponding actual values were designed. The experimental results are shown in Table 3. Overall, the range of T-AOC for the fermented samples was between 37.80 μmol/g and 18.96 μmol/g (containing 60.0% moisture) (Table 4).

Design-Expert 13.0 software was used to fit the response value of quercetin extraction by multiple linear regression. The quadratic polynomial regression equations were for the cellulase dosage (A), inoculation size (B), fermentation time (C), and T-AOC of the fermented MIP. The results for the model coefficient of significance and variance analysis are shown in Table 4. The regression model produced the following equation of the T-AOC as a function (Y) of cellulase dosage (A), inoculation size (B), and fermentation time (C):

(3) $$Y=35.5+1.97A+2.79B+1.42C-2.05AB-0.615AC+0.4986BC-4.5A^{2}-4.79B^{2}-6.73C^{2}$$

The data in Table 5 show that the coefficient of determination, R^2^ = 0.9672, adjusts the complex correlation coefficient R^2^_Adj_ = 0.9251, and the model misfit term *p* > 0.05. This shows that the model has high reliability. The quadratic regression equation obtained can fit the experimental data well and can be used to predict the impact of different fermentation conditions on the total antioxidant capacity of MIP. At the same time, the first term B and the second terms A^2^, B^2^, and C^2^ showed extremely significant performance, while the first terms A and C and the interaction term AB also showed significant performance. The optimized process parameters obtained by the Design-Expert 13.0 software are a cellulase dosage of 0.9%, an *L.f* ZC529 inoculation size of 4 mL/100 g, and a fermentation time of 7 days.

### 3.3. Interaction Effects of Different Experimental Factors on Response Variables

**Figure 2:**
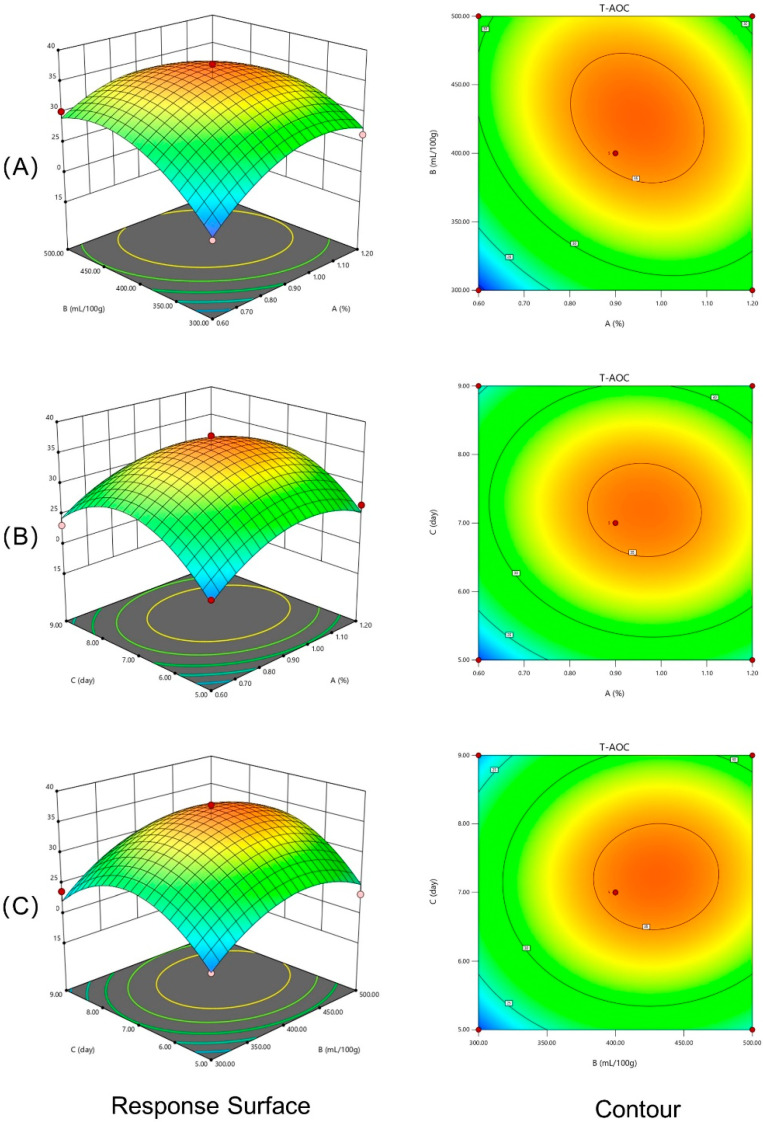
Response surface plots of the effect of factor interactions on the T-AOC of MIP. *Note*: (**A**) the effect of the interaction between cellulase dosage and *L.f* ZC529 inoculation size; (**B**) the effect of the interaction between cellulase dosage and fermentation time; and (**C**) the effect of the interaction between *L.f* ZC529 inoculation size and fermentation time.

Contour plots (2D) and response surface plots (3D) were generated from the regression Equation (3). The T-AOC as the responses to different cellulase dosages, inoculation size, and fermentation time are shown in Figure 2. The highest center of the surface diagram showed the extreme value for pairwise interactions. As shown in Figure 2A–C, the T-AOC first increases with the independent variables but then decreases during the experimental range. All the surfaces in Figure 2 were steep, indicating that the interactions among the cellulase dosage (A), inoculation size (B), and fermentation time (C) were very significant. The above results are consistent with the results of the variance analysis.

### 3.4. The Antioxidant Composition of Fermented MIP under the Optimum Conditions

**Figure 3:**
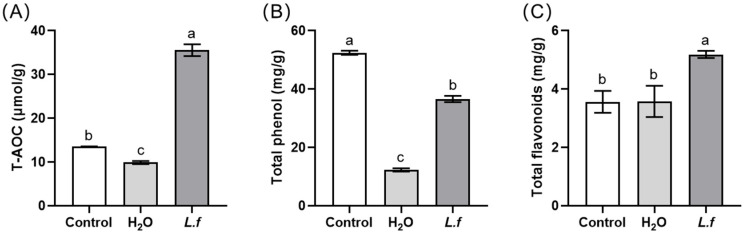
Antioxidant capacity and active components of MIP under different fermentation conditions. *Note:* (**A**): T-AOC, total antioxidant capacity; (**B**): total phenolic content; (**C**): total flavonoid content. Control group: unfermented MIP; *L.f* group: MIP was fermented under optimal conditions; H_2_O group: spontaneous fermentation, MIP was treated with sterile water of the same volume as *L.f* ZC529 inoculation size; Different lowercase letters indicate significant differences among treatment groups (*p* < 0.05).

Based on the optimal fermentation conditions, the changes in antioxidant capacity and content of antioxidant active components in MIP after 7 days of fermentation are shown in Figure 3. Compared to the control group, the T-AOC and total phenolic content of the H_2_O group were significantly reduced after 7 days (*p* < 0.05) (Figure 3A,B). After optimization of the fermentation process (*L.f* group), the T-AOC of MIP significantly increased (*p* < 0.05), with an increase of 160.72% (Figure 3A). Additionally, the total phenolic content in the *L.f* group was significantly higher than that in the H_2_O group (*p* < 0.05) (Figure 3B). And, the total flavonoid content in the *L.f* group was significantly higher than that in the control and H_2_O groups (*p* < 0.05) (Figure 3C).

### 3.5. In Vivo Irritation Properties of the Optimal Fermentation MIP Extract

**Figure 4:**
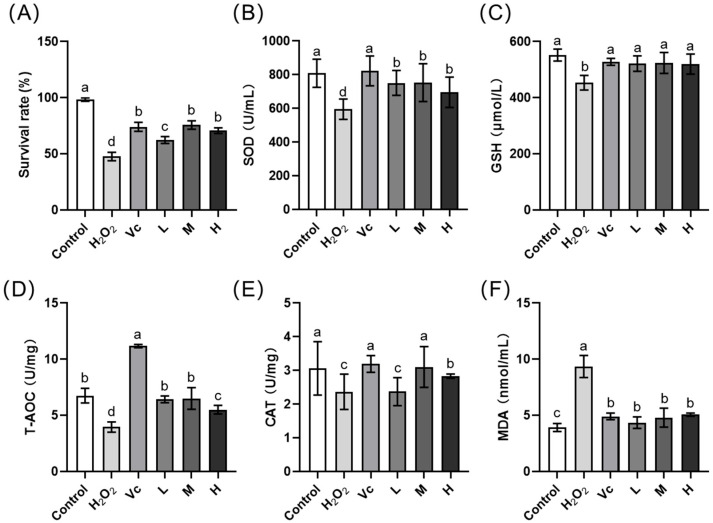
Effect of FMPIE on the antioxidative parameters in the oxidative stressed flies. *Note:* (**A**): survival rate; (**B**): SOD, superoxide dismutase; (**C**): GSH, glutathione; (**D**): T-AOC, total antioxidant capacity; (**E**): CAT, catalase; (**F**): MDA, malondialdehyde. The flies in the L, M, H, H_2_O_2_, and Vc groups were oxidative stress-induced flies. Control group: blank control; healthy flies fed with normal feed (a 5% sucrose solution); H_2_O_2_ group: negative control, normal feed; Vc group: positive control, fed with 0.5% Vc solution containing 5% sucrose; L group: low group, fed with 6.25% FMIPE containing 5% sucrose; M group: medium group, fed with 12.5% FMIPE containing 5% sucrose; H group: high group, fed with 25% FMIPE containing 5% sucrose. Different lowercase letters indicate significant differences among treatment groups (*p* < 0.05).

The effects of different concentrations of FMIPE on the survival rate of flies, MDA content in tissues, and activities of antioxidant stress-related enzymes are shown in Figure 4. Compared to the control group, H_2_O_2_ induction significantly reduced the survival rate of flies (*p* < 0.05). However, treatment with FMIPE at various concentrations significantly improved the survival rate (*p* < 0.05). When the extract concentration ranged from 12.5% to 25%, the protective effect on the survival rate was comparable to that of 0.5% Vc (*p* > 0.05) (Figure 4A). Compared to the negative control group, treatment with FMIPE at various concentrations significantly increased the GSH content, SOD activity, and T-AOC in flies (*p* < 0.05) (Figure 4C,D,F), while significantly decreasing the MDA content (*p* < 0.05) (Figure 4E). In the 12.5% and 25% treatment groups, the CAT activity was significantly increased (*p* < 0.05) (Figure 4B). Compared to the positive control, there was no difference in the GSH and MDA contents in the flies treated with FMIPE at various concentrations (*p* > 0.05) (Figure 4C,E). CAT activity in the 25% treatment group was consistent with the effect of Vc treatment (*p* > 0.05) (Figure 4B).

## 4. Discussion

During the fermentation process, microbial enzymatic catalysis can induce the release of phenolic compounds by breaking the bonds between phenolics and other components, leading to an increase in the antioxidant activity of fermentation products [24]. Most phenolic compounds exist primarily in conjugated forms linked to sugar groups and other compounds, such as organic acids, amines, and lipids, which reduce their ability to act as good antioxidants [25]. Carbohydrate-degrading enzymes produced by microorganisms during fermentation can hydrolyze these phenolic conjugates, releasing free phenolic substances from their inhibited conjugated forms and increasing the concentration of free phenolic compounds in the substrate [24,25]. Zhou et al. found that the content of free soy isoflavones and peptides in soy milk significantly increased after fermentation with *Lactobacillus fermentum* CQPC04, thereby enhancing the antioxidant capacity of soy milk [26]. Additionally, the fermentation of plant tissues by LAB can protect their bioactive substances. Pasquale Filannino et al. demonstrated the protective effect of LAB fermentation on the content of Vc and carotenoids in cactus cladodes (*Opuntia ficus-indica* L.) [27]. MIP, as agricultural waste, is estimated to generate approximately 16,800 tons of skin annually in Australia [2], with an annual production volume reaching 158,000 tons in China [1]. This study utilizes *L.f* ZC529 to ferment MIP, enhancing and preserving the antioxidant activity of MIP, which contributes to the better utilization and value addition of these waste materials. MIP has a high cellulose content and low availability of monosaccharides for *L.f* ZC529 utilization [1]. The addition of cellulase can degrade the cellulose and hemicellulose components in MIP into monosaccharides, promoting the proliferation of *L.f* ZC529 and shortening the fermentation time [28]. Additionally, phenolics may bind with cell-wall components, such as cellulose, lignin, and structural proteins, forming insoluble-bound phenolics (IBPs). Adding cellulase during fermentation can help facilitate the release of phenolics from IBPs [25]. Therefore, a synergistic fermentation approach combining bacteria and enzymes is adopted.

RSM is a statistical method used to simultaneously validate the effects and interactions of different parameters. Kuo et al. optimized the fermentation conditions using RSM to achieve the highest antioxidant activity in *Chenopodium formosanum* Koidz [18]. In this study, the RSM was used to optimize the synergistic fermentation conditions of MIP with cellulase and *L.f* ZC529. The optimal fermentation conditions were determined to be 0.9% cellulase dosage, 4 mL/100 g *L.f* ZC529 inoculation, and a fermentation time of 7 days. Under the optimal conditions, the T-AOC of the fermented product increased by 160.72% compared to before fermentation, and the total flavonoid content was also significantly higher than before fermentation. Moreover, the total phenolic content of MIP treated under optimal conditions was significantly higher than that of the H_2_O fermentation group. These results indicate that, under the above conditions, fermentation treatment of MIP can slow down the loss of total phenolic content and effectively increase the content and activity of its antioxidant components. Feng et al. pointed out that LAB produces antioxidant compounds, such as exopolysaccharides, carotenoids, ferulic acid, and histamine, during fermentation [29], and the production, concentration, and types of these antioxidant compounds are closely related to the fermentation substrate [30,31]. Moreover, the bacterial communities and metabolites in the fermentation system change with the fermentation environment [32]. In our experiment, the significant increase in the T-AOC of fermented MIP may also be related to the metabolites produced by *L.f*. Therefore, further research is needed to clarify the role of *L.f* metabolites and other bacterial activities in the enhanced antioxidant capacity of fermented MIP.

*Drosophila melanogaster*, as a classic model organism, offers advantages, such as small size, easy management, and low cost. Additionally, *Drosophila* shares 75% genomic similarity with humans [33]. Some studies have successfully evaluated the antioxidant capabilities of substances like royal jelly, tempol, and agar oligosaccharides using *Drosophila* [34,35,36]. Therefore, we chose *Drosophila* for further investigation in this study, and an oxidative stress *Drosophila^gstD-GFP^* model was prepared by H_2_O_2_, which was used to verify the in vivo antioxidant activity of fermented MIP. MDA, as a by-product of lipid peroxidation, has a concentration that is one of the most commonly used biomarkers reflecting the level of lipid peroxidation [37]. It has been reported that the fermentation of grape skin by *Lactobacillus fermentum* CQPC04 [38] and soy milk by *Lactiplantibacillus plantarum* LP95 can reduce MDA levels and inhibit the production of excess free radicals [39]. FMIPE exhibits high anti-lipid oxidation activity in vivo. In this study, flies under oxidative stress showed an increase in survival rate and a significant decrease in MDA levels compared to the negative control after feeding with various concentrations of FMIPE. 

Under excessive stress conditions, cells will produce numerous ROSs. Cells have evolved a balanced system to neutralize the extra ROSs [40], namely antioxidant systems that consist of enzymatic antioxidants, such as superoxide dismutase (SOD), catalase (CAT) [41,42], and glutathione peroxidases (GSH-Px), and thioredoxin (Trx), as well as non-enzymatic [43] antioxidants that collectively reduce the oxidative state. This study found that, after treatment with different doses of MIP extracts, the activities of antioxidant enzymes, such as T-AOC and SOD, as well as the GSH content in the flies, were significantly increased, indicating that MIP extracts can reduce oxidative damage to cells caused by free radicals. Furthermore, the moderate concentration of MIP extracts had a more significant effect on certain antioxidant enzyme activities, showing a dose-dependent effect. Compared to the positive control (Vc), MIP extracts showed similar effects in some aspects. *Drosophila* is a model organism with a very complex structure. Measuring the antioxidant capacity of a complex organism has certain limitations. Based on the above results, more precise cell and animal models can be employed in further research. However, the current findings already provide important evidence for the potential application of FMIPEs as natural antioxidants or a health supplement. 

Dulf et al. pointed out that increasing the exploitation and utilization value of agricultural and food by-products can effectively address pollution problems caused by insufficient collection and improper disposal of these wastes, and reduce biomass loss [24]. This study enhanced the antioxidant activity and content of antioxidant substances in MIP through microbial fermentation. This enhancement supports the application of MIP in the cosmetic, pharmaceutical, and feed industries [1,11]. The reintroduction of MIP by-products into the production chain not only provides an additional source of income but also helps to reduce solid-waste disposal problems, aligning with circular economy principles and sustainable development goals.

## 5. Conclusions

Through response surface optimization, the fermentation of MIP using *L.f* ZC529 and cellulase significantly increased the T-AOC, total phenolic, and flavonoid contents in MIP under the conditions of a cellulase dosage of 0.9%, an *L.f* ZC529 inoculation size of 4 mL/100 g, and a fermentation time of 7 days. As verified by an oxidative stress fruit-fly model, FMIPE significantly enhanced the activity of antioxidant enzymes in fruit flies and reduced oxidative damage induced by H_2_O_2_. This study effectively addressed the issue of antioxidant activity loss in MIP and provided a theoretical basis for the value-added and reuse of MIP.

## Figures and Tables

**Table 1:** Antioxidant components and content in MIP (dry matter bases).

Components	Content	References
tannins	17.53% ± 3.97 mg/g	[4]
total phenols	18.36 ± 0.32 mg/g	[5]
total flavonoids	7.33 ± 2.63 mg/g	[6]
4-hydroxybenzyl alcohol	1.3 ± 0.12 mg/g	[7]
3,4-dehydroxybenzoic acid	0.19 ± 0.001 mg/g	
p-hydroxybenzoic acid	0.046 ± 0.003 mg/g	
p-hydroxybenzaldehyde	0.15 ± 0.009 mg/g	

**Table 2:** Levels of three independent variables used in Box–Behnken design.

Factor	Coded Symbol	Level		
		−1	0	1
Cellulase dosage (%)	A	0.6	0.9	1.2
Inoculation size (mL/100 g)	B	3	4	5
Fermentation time (day)	C	5	7	9

**Table 3:** Grouping and treatment of *Drosophila*.

Flies	Groups	Treatment
H_2_O_2_-induced oxidative stress flies	Low group (L group)	fed with 6.25% FMIPE containing 5% sucrose
	Medium group (M group)	fed with 12.5% FMIPE containing 5% sucrose
	High group (H group)	fed with 25% FMIPE containing 5% sucrose
	H_2_O_2_ group	negative control, feed with 5% sucrose solution (normal feed)
	Vc group	positive control, fed with 0.5% Vc solution containing 5% sucrose
Healthy flies	Control group	blank control, feed with 5% sucrose solution (normal feed)

**Table 4:** Box–Behnken experimental design and response for the entrapment efficiency.

Experimental No.	A	B	C	T-AOC (μmol/g)
1	−1	0	1	23.18
2	0	0	0	35.27
3	0	0	0	35.38
4	1	0	1	26.51
5	0	1	−1	23.26
6	0	−1	1	23.71
7	0	1	1	28.76
8	0	−1	−1	20.20
9	−1	−1	0	18.96
10	0	0	0	34.21
11	0	0	0	37.80
12	−1	0	−1	20.79
13	0	0	0	34.86
14	−1	1	0	30.15
15	1	0	−1	26.58
16	1	1	0	29.37
17	1	−1	0	26.36

**Table 5:** ANOVA of the central composite design.

Source	Sum of Squares	df	Mean Squares	*F*-Value	*p*-Value	Significance
Model	543.21	9	60.36	22.95	0.0002	**
A	30.98	1	30.98	11.78	0.0110	*
B	62.29	1	62.29	23.68	0.0018	**
C	16.05	1	16.05	6.10	0.0428	*
AB	16.75	1	16.75	6.37	0.0396	*
AC	1.51	1	1.51	0.5753	0.4729	
BC	0.9943	1	0.9943	0.3780	0.5581	
A^2^	85.38	1	85.38	32.46	0.0007	**
B^2^	96.52	1	96.52	36.70	0.0005	**
C^2^	190.94	1	190.94	72.60	<0.0001	**
Residual	18.41	7	2.63			
Lack of Fit	10.99	3	3.66	1.98	0.2597	
Pure Error	7.42	4	1.85			
Cor total	561.62	16				
R^2^	0.9672					
R^2^_Adj_	0.9251					

*Note:* ** Highly significant (*p* < 0.01), * significant (*p* < 0.05), and ns for not significant.

## Data Availability

The original contributions presented in the study are included in the article, further inquiries can be directed to the corresponding authors.
